# Partial-Hepatectomized (70%) Model Shows a Correlation between Hepatocyte Growth Factor Levels and Beta-Cell Mass

**DOI:** 10.3389/fendo.2015.00020

**Published:** 2015-02-16

**Authors:** Tiago G. Araújo, Alexandre G. Oliveira, Mario J. A. Saad

**Affiliations:** ^1^Department of Internal Medicine, State University of Campinas (UNICAMP), Campinas, Brazil; ^2^Department of Physiology and Pharmacology, Federal University of Pernambuco (UFPE), Recife, Brazil; ^3^Department of Physical Education, São Paulo State University (UNESP), Rio Claro, Brazil

**Keywords:** liver regeneration, hepatectomy, beta-cell, pancreas, HGF

One of the best-known Greek myths of all time is the tragedy of the Titan Prometheus, who stole fire from Olympus to deliver it to the human race. Furious, Zeus demands that Prometheus is chained to a rock at the peak of the Caucasus and condemns him to have the liver eternally devoured by an eagle. Every time that the eagle finished eating the liver from Prometheus, the liver was regenerated and the eagle began all over again. Then, it is not today what science knows the liver’s ability to regenerate and its importance for the human body.

It is known that the liver and the pancreas are derived embryonically from the gut entoderm, then, following this line of reasoning is not surprising to formulate the hypothesis of a possible strong connection between the liver and the pancreas. In this sense, during the years 1986–1993, researchers have been demonstrated that liver regeneration is accompanied by pancreatic growth in different species of animals, such as golden hamsters (1), dogs (2), and rats (3). However, further studies to characterize the possible factors and mechanisms behind this connection (liver regeneration → effects on the pancreas) were needed.

Recently, Moreau et al. (4) described the early effects of liver regeneration on the endocrine pancreas. They utilized a traditional model of liver regeneration in rodents called the “Higgins procedure,” which consists of a 70% hepatectomy (termed 2/3 partial hepatectomy) (5). This was done to evaluate the role of the systemic factors released throughout the 72 h after the surgery, based on the structure and function of the endocrine pancreas. Then, in this assessment of the time-course evolution, they observed that the relative pancreas weight was higher in hepatectomized rats when compared to that of the sham-operated rats. On the other hand, no significant increase in beta-cell proliferation was detected 3 days after surgery. Furthermore, by performing the measurement of possible factors released by the liver 3 days after the procedure, no difference in serum hepatocyte growth factor (HGF) levels was observed. However, there was a trend toward a significant increase in the serum VEGF levels and a significant increase in the microparticle levels. Additionally, in an *in vitro* experiment using RIN-m5F cells, they observed that the sera obtained from animals 3 days after hepatectomy was more efficient in maintaining the viability of this mentioned linage (4).

Our laboratory in recent times investigated the role of HGF in insulin resistance-associated compensatory mechanisms (6). As a tool of this study, we used the 70% hepatectomy model that shows an increase of endogenous HGF (7, 8), which represents the major mechanism that controls hepatic cell proliferation and regeneration (7, 8). In our study, we observed the temporal evolution of fasting plasma HGF and insulin levels beyond the β-cell mass, as well as the beta-cell proliferation (percentage of Ki-67-positive cell in β-cells) in partial-hepatectomized (70%) and sham-operated rats over the course of the experiment’s 7 days. In agreement with the research by Moreau et al. (4), our study previously demonstrated that during the first 3 days after surgery, the circulating HGF levels from hepatectomized rats are similar to those in the sham group. However, from the fourth day, the HGF levels from the hepatectomized group rose and remained high until the seventh day of the experiment. In parallel, this augmentation was followed by a significant increase in the islet mass, together with augmentation on the Ki-67-positive nuclei of the β-cells on the seventh day. As a result, we also observed an increase in the plasma insulin level from the fifth day, which remained high until the seventh day after the procedure (6).

It is noteworthy that our data corroborated and complemented the results presented by Moreau et al. (4). We can observe that in a longer study (7 days), the endocrine pancreas’ responses to the early effects of liver regeneration become more evident. In addition, the effect of HGF on the islets is very clear to us and has been proven through both our data (6) and other pieces of evidence in the literature (9–11).

One of the attractive aspects of this liver-pancreas axis is related to the current clinical islet transplantation. In this way, the studies discussed here show a significant importance for clinical practice, because recently a study by Saito et al. (12) showed that partial hepatectomy can improve the intraportal islet transplantation in rats. In a further discussion about this study, it is worth to mention that after partial hepatectomy procedure, many growth factors such as HGF and VEGF are released in the circulation during the hepatic regeneration (7, 8, 12). These factors are well known to have properties to promote vascularization (12). Therefore, this study by Saito et al. demonstrated that partial hepatectomy improved the outcome of intraportal islet transplantation by the revascularization of the transplanted islets. In this regard, it is possible to propose that the induction of hepatic regeneration might be one of the promising strategies to improve the islet transplantation process.

In conclusion, it is important to note that due to the relevance of this issue to scientific community as presented in this letter, the discussion of the differences observed in the works from Araújo et al. (6) and Moreau et al. (4) is a matter of great concern. Thus, although we think that the research by Moreau et al. (4) is a significant contribution to the studies on the liver–pancreas axis, we believe that these results should have been considered in the context of earlier published investigations.

## Conflict of Interest Statement

The authors declare that the research was conducted in the absence of any commercial or financial relationships that could be construed as a potential conflict of interest.

## References

[1] Rao MS Subbarao V. DNA synthesis in exocrine and endocrine pancreas after partial hepatectomy in Syrian golden hamsters. Experientia (1986) 42(7):833–4. 10.1007/BF01941543 3525211

[2] Hirano T Manabe T Tobe T. Morphological and functional discrepancies in endocrine pancreas after partial hepatectomy in dogs. HPB Surg (1991) 3(2):103–14. 10.1155/1991/19038 2043508 PMC2423602

[3] Printz H Goke B Fehmann HC Weiershausen S Nustede R Neurath M Partial hepatectomy affects pancreatic size and function in rats. Pancreas (1993) 8(2):233–9. 10.1097/00006676-199303000-00015 7681582

[4] Moreau F Seyfritz E Toti F Sigrist S Bietigier W Pinget M Early effects of liver regeneration on endocrine pancreas: in vivo change in islet morphology and in vitro assessment of systemic effects on beta-cell function and viability in the rat model of two-thirds hepatectomy. Horm Metab Res (2014) 46(13):921–6. 10.1055/s-0034-1389995 25376550

[5] Higgins GM Anderson RM Experimental pathology of the liver I. Restoration of the liver of the white rat following surgical removal. Arch Pathol (1931) 12(4):186–206.

[6] Araújo TG Oliveira AG Carvalho BM Guadagnini D Protzek AO Carvalheira JB Hepatocyte growth factor plays a key role in insulin resistance-associated compensatory mechanisms. Endocrinology (2012) 153(12):5760–9. 10.1210/en.2012-1496 23024263

[7] Michalopoulos GK. Liver regeneration. J Cell Physiol (2007) 213(2):286–300. 10.1002/jcp.21172 17559071 PMC2701258

[8] Michalopoulos GK. Liver regeneration after partial hepatectomy: critical analysis of mechanistic dilemmas. Am J Pathol (2010) 176(1):2–13. 10.2353/ajpath.2010.090675 20019184 PMC2797862

[9] Garcia-Ocana A Takane KK Syed MA Philbrick WM Vasavada RC Stewart AF. Hepatocyte growth factor overexpression in the islet of transgenic mice increases beta cell proliferation, enhances islet mass, and induces mild hypoglycemia. J Biol Chem (2000) 275(2):1226–32. 10.1074/jbc.275.2.1226 10625667

[10] Dai C Huh CG Thorgeirsson SS Liu Y. Beta-cell-specific ablation of the hepatocyte growth factor receptor results in reduced islet size, impaired insulin secretion, and glucose intolerance. Am J Pathol (2005) 167(2):429–36. 10.1016/S0002-9440(10)62987-2 16049329 PMC1603568

[11] Araújo TG Oliveira AG Saad MJ Insulin-resistance-associated compensatory mechanisms of pancreatic beta cells: a current opinion. Front Endocrinol (2013) 4:146 10.3389/fendo.2013.00146 PMC3796265 24133484

[12] Saito Y Chan NK Hathout E. Partial hepatectomy improves the outcome of intraportal islet transplantation by promoting revascularization. Islets (2012) 4(2):138–44. 10.4161/isl.19491 22622159 PMC3396702

